# Three Questions to Consider Before Applying Ecological Momentary Interventions (EMI) in Psychiatry

**DOI:** 10.3389/fpsyt.2020.00333

**Published:** 2020-04-16

**Authors:** Marthe Gründahl, Jürgen Deckert, Grit Hein

**Affiliations:** Translational Social Neuroscience Unit, Center of Mental Health, Department of Psychiatry, Psychosomatic and Psychotherapy, University of Würzburg, Würzburg, Germany

**Keywords:** ecological momentary intervention, mHealth, psychiatric disorders, randomized controlled trials, face-to-face therapy, physiology

## Introduction

The umbrella term Ecological Momentary Intervention (EMI) labels a group of methods that provide clinical treatment *via* mobile devices in individuals’ daily lives. The development and application of EMI are continuously increasing (1–3), related to the emergence of smartphone apps and the widespread availability of the internet (4, 5). Today, EMI applications are offered as treatment of various different psychological and psychiatric disorders including anxiety disorders, depression, obsessive-compulsive disorders, and posttraumatic stress disorders (2, 4, 6). EMI are often based on clinically evaluated psychotherapeutic treatments, e.g., cognitive behavioral therapy (CBT) (2), and apply therapeutic activities, assignments, and skills outside the clinical/therapeutic setting, i.e., in individuals’ natural environment (7, 8). There are estimates that 70–85% of US citizens in need of mental health care do not receive it, and that these numbers might even be higher worldwide (9). EMI has the potential to reduce this treatment gap by providing interventions to individuals with little or no prospects of receiving regular clinical, face-to-face treatment. By providing wider access to mental health care, EMI may have financial benefits, potentially reducing the need for clinical care and hospitalization. Finally, EMI allows clinicians to monitor and individualize a therapeutic intervention, with patients actively included in the treatment process, thus resulting in higher treatment compliance and better interaction between patients and clinicians (10).

With all the potential benefits of EMI in mind, we propose that clinicians should ask the following three questions before integrating EMI into the therapy of psychological and psychiatric disorders. 1) Is the efficacy of the EMI application proven by randomized controlled trials, as per the criterion for all accredited therapies (especially those covered by health insurance)? 2) Does the EMI application consolidate the effects of face-to-face therapy, for example by reducing the risk of relapse? 3) Does the EMI application provide additional measures of therapy outcomes that are more accurate than traditional evaluation methods (e.g., clinical interviews and questionnaires)?

## Evidence From Randomized Controlled Trials

Randomized controlled trials (RCT) are seen as the gold standard in medical research that should be applied to all treatment methods to prove their efficacy [(11, 12) but see Grossman and Mackenzie (13) for a critical approach]. In RCTs, patients are randomly assigned to an experimental group that receives a certain treatment, or one or more control groups that receive a placebo or no treatment. The power of the conclusions gained from an RCT depends on the quality of the placebo treatment. In the ideal case, the control group receives a non-potent treatment that otherwise is identical to the experimental group. Regarding EMI applications, most RCT studies compared experimental groups that receive EMI treatment [usually combined with “therapy as usual [TAU]” or at least weekly face-to-face feedback (14)] and control groups that only receive TAU (15, 16).

A recent systematic review of EMI applications for major depressive disorder (MDD) by Colombo et al. (5) included seven EMI studies. The authors argue in favor of EMI’s clinical efficacy and feasibility in treating MDD. However, the review only included four different EMI, and only two RCTs on their efficacy (5). Thus, the generalizability of these findings is questionable. A meta-analysis by Linardon et al. (4) included more data and a statistical analysis of effect sizes. Based on 66 RCTs of mental health EMI applications in clinical and non-clinical samples, they reported a reduction of stress and depressive and anxiety symptoms with small to medium effects (Hedge’s *g* between 0.28 and 0.58). Such evidence suggests that EMI applications can indeed have systematic effects on important aspects of mental health (4). Given that they are effective, it is now crucial to understand which components of the EMI drive the effect. Most of the existing popular EMI applications for depression and anxiety include a variety of treatment components such as psychoeducation, assessment, relaxation, mindfulness, and meditation (17). Some of the individual components have been proven effective in isolation, but it is unclear if they drive the overall effect of the EMI application and how they interact with the other components.

To understand the mechanism behind EMI effects, RCTs need to systematically test the effect of individual components against each other. For example, Burton et al. (15) found a reduction of depression symptoms through regular use of an EMI application (“Help4Mood”) in which patients interact with a customized virtual agent that guides them through daily mood questionnaires (15). To specify this effect, a group of patients using the current EMI version (virtual agent plus daily mood reflection) should be compared with patients that interact with the same virtual agent, but answer neutral questions that are unrelated to psychiatric symptomes.

Notably, it has been suggested that RCTs may not be the gold standard for the evaluation of increasingly smartphone-based EMI (3) because of their long duration and high financial costs (6, 18). However, given that a successful evaluation in RCTs is required before the official release of other medical treatments, these standards should also be applied to EMI.

## Evidence for Consolidation of Face-to-Face Therapy Effects

Face-to-face therapy is efficient in treating current psychiatric symptoms, for example in reducing depression or anxiety at the time of the consultation. However, maintaining these effects and preventing relapse is challenging (19). Because they can be integrated into patients’ everyday life, EMI applications have the potential to consolidate face-to-face treatment effects, to prevent relapse and thus to increase the periods between face-to-face consultation (4, 7, 20). Up to this point, evidence for the efficacy of EMI in preserving face-to-face therapy effects is scarce (21–23).

There are some follow-up investigations on internet-based and smartphone-based EMI (4). For instance, a review by Andersson et al. (21) investigated 14 studies (*n* = 902) on internet-based, guided EMI with Cognitive Behavioral Therapy (CBT) elements for mental health improvement, with follow-up assessments between 2 and 5 years. They reported an average 50% symptom improvement from baseline and large within-group effects, with a pre- to follow-up effect size of Hedge’s *g* = 1.52. However, the included studies and their effect sizes were heterogeneous, and overall results might thus be overestimated. Välimäki et al. (20) investigated effects of internet-based EMI on stress, depression, and anxiety in youth. Fifteen RCTs (*n* = 4979) were included into meta-analysis. Results suggest short-term improvement of mental health and reduction of depression and anxiety, but were questionable regarding long-term effectiveness due to a limited number of follow-up assessments. Only two studies reported significant long-term (>6 months) improvement of depression symptoms, while no mid- or long-term effects were evident for anxiety, mood, and feelings (20). More follow-up assessments are needed.

Long-term effects of EMI apps have mainly been assessed in relatively short follow-up periods of 4 weeks to 3 months (4, 24). However, there are single studies with promising long-term results. A small study on the combination of a well-being app and face-to-face counseling in university students (*n* = 38) with moderate anxiety or depression showed clinically relevant reductions in symptoms that were still evident after 6 months (25). Economides et al. (19) investigated long-term effects of an EMI app (“Ascend”) with evidence-based treatment components in 102 patients. They found anxiety-reducing effects to last up to 6 months (Hedge’s *g* = 0.91), and depressive-reducing effects to last up to 12 months after the initial 8-week EMI treatment (Hedge’s *g* = 1.14). Notably, these results show uncontrolled effect sizes and would have greatly profited from the comparison to a control group, i.e., an RCT.

Overall, the length of follow-up periods varies greatly and is often considered too short (4, 21, 26), resulting in a lack of sufficient evidence on EMI long-term treatment effects. Moreover, there is a lack of longitudinal studies that compare the effects of face-to-face treatments with the effects of the same face-to-face treatments in combination with EMI.

## Optimization of Therapy Outcome Measures

Currently, the efficacy of EMI applications is mostly evaluated based on patients’ self-reports and thus not different from the evaluation of classical face-to-face therapies. In most cases, questionnaires or reports on mental health outcomes are filled in prior to and after the intervention (e.g., 27, 28), sometimes with additional follow-up assessments (e.g., 19, 29). While self-reports are necessary to gain insights into the subjective perception and evaluation of symptoms, they are potentially confounded by biases (30). For example, there is a response bias in favor of positive responses (i.e., acquiescence, saying “yes”) compared to negative responses (saying “no”; 31, 32). Moreover, patients may feel obliged to indicate a reduction of symptoms after the EMI to justify their own effort or to please the clinician (e.g., 33, 34).

It is well known that the onset and symptomology of mental health disorders like depression and anxiety are associated with various biomarkers (35). For example, MDD is associated with decreased high frequency heart rate variability (HF-HRV), both at rest and in response to challenges (36); increased cortisol levels during stress recovery (37); and decreases in skin conductance (38). Anxiety disorders, too, are associated with decreased HF-HRV (36). They also relate to high sensitivity of skin conductance to general anxiety change. Phobias are particularly associated with high cardiovascular activity (38). In combination with self-reports, these and other biomarkers can predict the onset and severity of psychiatric conditions (5, 39), and thus should be used to verify therapy outcomes.

A small-sampled clinical EMI study on stress and anger management in veterans (27) used mobile cardiovascular and electrodermal measurements to indicate physiological stress. Both the control group (*n* = 6) and the experimental group (*n* = 10) received standard CBT for 8 to 10 weeks. An EMI app with physiological stress assessment (skin conductance, heart rate, cortisol) was integrated into the experimental group’s CBT. If stress was detected, the app initiated an alert and presented exercises for stress reduction. Results suggest that the combined vs standard intervention was more efficient in reducing stress, anxiety, and anger. Notably, however, only 4 experimental and 3 control participants completed follow-up assessments (27).

Botella et al. (40) investigated the feasibility and efficacy of an internet-based CBT-EMI for the prevention and treatment of depression and adjustment disorders in 60 unemployed men at risk of developing depression. The sensor group (*n* = 19) received 6 to 10 weeks of EMI with additional physiological and activity sensor assessment (electrocardiography, electroencephalography, and actigraphy) providing graphic feedback. The intervention group (*n* = 22) received EMI without sensors. The control group (*n* = 19) received no treatment. Overall, EMI related to stronger improvement in clinical variables. This effect was more pronounced in the sensor group, with medium effect sizes for depression, affect, and stress, and a small effect size for anxiety. The intervention group showed small effect sizes for all outcomes except depression (medium). In sum, EMI allows the development of physiological and behavioral outcome parameters in addition to classical psychometric scores. The combination of EMI and physiological measures is thus promising in MDD, anxiety, and beyond, but its potential and risks as well as ethical aspects need further investigation (41).

## Conclusion

In summary, there is mounting evidence *via* randomized controlled trials of the efficacy of EMI applications, but a lack of evidence that EMI applications consolidate face-to-face treatment effects, for example, prevention of relapse. Moreover, the EMIs have not yet reached their full potential in providing more objective therapy outcome measures, for example by systematically assessing physiological changes in the course of the EMI. In the light of these findings, we answer our first question (Evidence from randomized controlled trials)? with a cautious “Yes,” and our second and third questions (Evidence for consolidation of face-to-face therapy?/Optimization of therapy outcome measures)? with an optimistic “Not yet.” Given the intense research activities in the field, the state of EMI evidence regarding our three questions should be regularly reviewed and recommendations for their application in the clinical praxis should be updated.

## Author Contributions

MG and GH wrote the manuscript. JD amended the manuscript.

## Funding

This article was supported by the German Research Foundation (HE 4566/5-1).

## Conflict of Interest

JD is involved in studies with P1Vital and BioVariance on medical device development financed by the European Union Horizon 2020 SME grant and a regional development and energy grant of the Bavarian Ministry of Economic Affairs respectively.

The remaining authors declare that the research was conducted in the absence of any commercial or financial relationships that could be construed as a potential conflict of interest.

## References

[1] Myin-Germeys I Kasanova Z Vaessen T Vachon H Kirtley O Viechtbauer W Experience sampling methodology in mental health research: new insights and technical developments. World Psychiatry (2018) 17(2):123–32. 10.1002/wps.20513 PMC5980621 29856567

[2] Van Ameringen M Turna J Khalesi Z Pullia K Patterson B There is an app for that! The current state of mobile applications (apps) for DSM-5 obsessive-compulsive disorder, posttraumatic stress disorder, anxiety and mood disorders. Depress Anxiety (2017) 34(6):526–39. 10.1002/da.22657 28569409

[3] Marshall JM Dunstan DA Bartik W Clinical or gimmickal: The use and effectiveness of mobile mental health apps for treating anxiety and depression. Aust N Z J Psychiatry (2020) 54(1):20–8. 10.1177/0004867419876700 31552747

[4] Linardon J Cuijpers P Carlbring P Messer M Fuller-Tyszkiewicz M The efficacy of app-supported smartphone interventions for mental health problems: a meta-analysis of randomized controlled trials. World Psychiatry (2019) 18(3):325–36. 10.1002/wps.20673 PMC6732686 31496095

[5] Colombo D Fernandez-Alvarez J Patane A Semonella M Kwiatkowska M Garcia-Palacios A Current State and Future Directions of Technology-Based Ecological Momentary Assessment and Intervention for Major Depressive Disorder: A Systematic Review. J Clin Med (2019) 8(4):465. 10.3390/jcm8040465 PMC6518287 30959828

[6] Schueller SM Aguilera A Mohr DC Ecological momentary interventions for depression and anxiety. Depression Anxiety (2017) 34(6):540–5. 10.1002/da.22649 28494123

[7] Heron KE Smyth JM Ecological momentary interventions: Incorporating mobile technology into psychosocial and health behaviour treatments. Brit J Health Psych (2010) 15:1–39. 10.1348/135910709x466063 PMC2800172 19646331

[8] Versluis A Verkuil B Spinhoven P van der Ploeg MM Brosschot JF Changing Mental Health and Positive Psychological Well-Being Using Ecological Momentary Interventions: A Systematic Review and Meta-analysis. J Med Internet Res (2016) 18(6):e152. 10.2196/jmir.5642 27349305 PMC4940607

[9] Kazdin AE Annual Research Review: Expanding mental health services through novel models of intervention delivery. J Child Psychol Psychiatry (2019) 60(4):455–72. 10.1111/jcpp.12937 29900543

[10] Gaggioli A Riva G From mobile mental health to mobile wellbeing: opportunities and challenges. In: Stud Health Technol Inform. (2013) 184:141–7. 10.1037/e574802013-193 23400146

[11] Nyström L Wall S Rutqvist L Lindgren A Lindqvist M Rydén S Breast cancer screening with mammography: overview of Swedish randomised trials. Lancet (1993), 341(8851):973–8. 10.1016/0140-6736(93)91067-V 8096941

[12] Stolberg HO Norman G Trop I Fundamentals of clinical research for radiologists. AJR (2004) 183:1539–44. 10.2214/ajr.183.6.01831539 15547188

[13] Grossman J Mackenzie FJ The randomized controlled trial: gold standard, or merely standard? Perspect Biol Med (2005) 48(4):516–34. 10.1353/pbm.2005.0092 16227664

[14] Kramer I Simons CJ Hartmann JA Menne-Lothmann C Viechtbauer W Peeters F A therapeutic application of the experience sampling method in the treatment of depression: a randomized controlled trial. World Psychiatry (2014) 13(1):68–77. 10.1002/wps.20090 24497255 PMC3918026

[15] Burton C Szentagotai Tatar A McKinstry B Matheson C Matu S Moldovan R Pilot randomised controlled trial of Help4Mood, an embodied virtual agent-based system to support treatment of depression. J Telemed Telecare (2016) 22(6):348–55. 10.1177/1357633X15609793 26453910

[16] Dahne J Lejuez CW Diaz VA Player MS Kustanowitz J Felton JW Pilot Randomized Trial of a Self-Help Behavioral Activation Mobile App for Utilization in Primary Care. Behav Ther (2019) 50(4):817–27. 10.1016/j.beth.2018.12.003 PMC6582985 31208690

[17] Wasil AR Venturo-Conerly KE Shingleton RM Weisz JR A review of popular smartphone apps for depression and anxiety: Assessing the inclusion of evidence-based content. Behav Res Ther (2019) 123:103498. 10.1016/j.brat.2019.103498 31707224

[18] Pham Q Wiljer D Cafazzo JA Beyond the Randomized Controlled Trial: A Review of Alternatives in mHealth Clinical Trial Methods. JMIR Mhealth Uhealth (2016) 4(3):e107. 10.2196/mhealth.5720 27613084 PMC5035379

[19] Economides M Ranta K Nazander A Hilgert O Goldin PR Raevuori A Long-Term Outcomes of a Therapist-Supported, Smartphone-Based Intervention for Elevated Symptoms of Depression and Anxiety: Quasiexperimental, Pre-Postintervention Study. JMIR Mhealth Uhealth (2019) 7(8):e14284. 10.2196/14284 31452521 PMC6733157

[20] Välimäki M Anttila K Anttila M Lahti M Web-Based Interventions Supporting Adolescents and Young People With Depressive Symptoms: Systematic Review and Meta-Analysis. JMIR Mhealth Uhealth (2017) 5(12):e180. 10.2196/mhealth.8624 29222079 PMC5741826

[21] Andersson G Rozental A Shafran R Carlbring P Long-term effects of internet-supported cognitive behaviour therapy. Expert Rev Neurother (2018) 18(1):21–8. 10.1080/14737175.2018.1400381 29094622

[22] Lindhiem O Bennett CB Rosen D Silk J Mobile technology boosts the effectiveness of psychotherapy and behavioral interventions: a meta-analysis. Behav Mod (2015) 39(6):785–804. 10.1177/0145445515595198 PMC4633319 26187164

[23] Newman MG Przeworski A Consoli AJ Taylor CB A randomized controlled trial of ecological momentary intervention plus brief group therapy for generalized anxiety disorder. Psychotherapy (2014) 51(2):198. 10.1037/a0032519 24059730 PMC4440457

[24] Marshall JM Dunstan DA Bartik W The Digital Psychiatrist: In Search of Evidence-Based Apps for Anxiety and Depression. Front Psychiatry (2019) 10:831. 10.3389/fpsyt.2019.00831 31803083 PMC6872533

[25] Broglia E Millings A Barkham M Counseling With Guided Use of a Mobile Well-Being App for Students Experiencing Anxiety or Depression: Clinical Outcomes of a Feasibility Trial Embedded in a Student Counseling Service. JMIR Mhealth Uhealth (2019) 7(8):e14318. 10.2196/14318 31418424 PMC6714497

[26] Tonning ML Kessing LV Bardram JE Faurholt-Jepsen M Methodological Challenges in Randomized Controlled Trials on Smartphone-Based Treatment in Psychiatry: Systematic Review. J Med Internet Res (2019) 21(10):e15362. 10.2196/15362 31663859 PMC6914239

[27] Winslow BD Chadderdon GL Dechmerowski SJ Jones DL Kalkstein S Greene JL Development and Clinical Evaluation of an mHealth Application for Stress Management. Front Psychiatry (2016) 7:130. 10.3389/fpsyt.2016.00130 27507949 PMC4960497

[28] LaFreniere LS Newman MG. A Brief Ecological Momentary Intervention for Generalized Anxiety Disorder: A Randomized Controlled Trial of the Worry Outcome Journal. Depress Anxiety (2016) 33(9):829–39. 10.1002/da.22507 27062682

[29] Ben-Zeev D Buck B Chu PV Razzano L Pashka N Hallgren KA Transdiagnostic Mobile Health: Smartphone Intervention Reduces Depressive Symptoms in People With Mood and Psychotic Disorders. JMIR Ment Health (2019) 6(4):e13202. 10.2196/13202 30977736 PMC6484257

[30] Trull TJ Ebner-Priemer U The Role of Ambulatory Assessment in Psychological Science. Curr Dir Psychol Sci (2014) 23(6):466–70. 10.1177/0963721414550706 PMC4269226 25530686

[31] Furnham A Henderson M The good, the bad and the mad: Response bias in self-report measures. Pers Individ Dif (1982) 3(3):311–20. 10.1016/0191-8869(82)90051-4

[32] Paulhus D. L "Measurement and control of response bias". In: Robinson JP Shaver PR Wrightman LS editors. Measures of personality and social psychological attitudes. San Diego, CA: Academic Press (1991). p. 17–59. 10.1016/B978-0-12-590241-0.50006-X

[33] Van de Mortel TF Faking it: social desirability response bias in self-report research. Aust J Advanced Nursing (2008) 25(4):40 Available at https://www.ajan.com.au/archive/ajan_25.4.html.

[34] Adams AS Soumerai SB Lomas J Ross-Degnan D Evidence of self-report bias in assessing adherence to guidelines. Int J Qual Health Care (1999) 11(3):187–92. 10.1093/intqhc/11.3.187 10435838

[35] Allen AP Kennedy PJ Cryan JF Dinan TG Clarke G Biological and psychological markers of stress in humans: Focus on the Trier Social Stress Test. Neurosci Biobehav R (2014) 38:94–124. 10.1016/j.neubiorev.2013.11.005 24239854

[36] Beauchaine TP Thayer JF Heart rate variability as a transdiagnostic biomarker of psychopathology. Int J Psychophysiol (2015) 98(2):338–50. 10.1016/j.ijpsycho.2015.08.004 26272488

[37] Burke HM Davis MC Otte C Mohr DC Depression and cortisol responses to psychological stress: a meta-analysis. Psychoneuroendocrinology (2005) 30(9):846–56. 10.1016/j.psyneuen.2005.02.010 15961250

[38] Boucsein W Electrodermal activity. (2nd ed.). New York, NY: Springer (2012). 10.1007/978-1-4614-1126-0

[39] Dogan E Sander C Wagner X Hegerl U Kohls E Smartphone-Based Monitoring of Objective and Subjective Data in Affective Disorders: Where Are We and Where Are We Going? Systematic Review. J Med Internet Res (2017) 19(7):e262. 10.2196/jmir.7006 28739561 PMC5547249

[40] Botella C Mira A Moragrega I Garcia-Palacios A Breton-Lopez J Castilla D An Internet-based program for depression using activity and physiological sensors: efficacy, expectations, satisfaction, and ease of use. Neuropsychiatr Dis Treat (2016) 12:393–406. 10.2147/NDT.S93315 27042067 PMC4770071

[41] Seppälä J De Vita I Jamsa T Miettunen J Isohanni M Rubinstein K Mobile Phone and Wearable Sensor-Based mHealth Approaches for Psychiatric Disorders and Symptoms: Systematic Review. JMIR Ment Health (2019) 6(2):e9819. 10.2196/mental.9819 30785404 PMC6401668

